# Palbociclib: a first-in-class CDK4/CDK6 inhibitor for the treatment of hormone-receptor positive advanced breast cancer

**DOI:** 10.1186/s13045-015-0194-5

**Published:** 2015-08-13

**Authors:** Janice Lu

**Affiliations:** Division of Hematology/Oncology, Department of Medicine, David Geffen School of Medicine, University of California, Los Angeles, CA USA

**Keywords:** Breast cancer, CDK4/CDK6 inhibitor, Palbociclib

## Abstract

Palbociclib was approved by the FDA for use in combination with letrozole for the treatment of postmenopausal women with hormone-receptor-positive, HER2-negative advanced breast cancer as initial endocrine-based therapy. In addition, the combination of palbociclib with fulvestrant resulted in superior outcome than fulvestrant alone in those who had progressed during prior endocrine therapy. This research highlight summarized the current development of CDK4/CDK6 inhibitors and future directions in the treatment of advanced hormone-receptor-positive breast cancer.

Personalizing the use of cancer therapeutics is a major focus of current cancer research [1–6]. The pioneering work of Finn and Slamon showed activity of palbociclib as an inhibitor of cyclin-dependent kinase (CDK) 4 and 6 which reduced cellular proliferation of estrogen receptor (ER)-positive breast cancer cell lines by blocking progression of cells from G1 into S phase of the cell cycle [7]. Palbociclib was approved by the FDA for use in combination with letrozole for the treatment of postmenopausal women with ER-positive, human epidermal growth factor receptor 2 (HER2)-negative advanced breast cancer as initial endocrine-based therapy for their metastatic disease.

The approval of palbociclib is based on a phase 2 PALOMA-1/TRIO-18 study, which is a randomized, multicenter, open-label trial in postmenopausal women with HR-positive, HER2-negative, advanced breast cancer who had not received previous systemic treatment for advanced disease. The trial enrolled 165 patients randomly allocated to receive either palbociclib plus letrozole or letrozole alone [8]. Among the 165 patients, 43 % had received chemotherapy and 33 % had received anti-hormonal therapy as a neoadjuvant or adjuvant treatment. Forty-nine percent of patients had no prior systemic therapy in the neoadjuvant or adjuvant setting. The majority of patients (98 %) had metastatic disease, 48 % had visceral disease, 75 % had bone disease, and 19 % had bone-only disease. Median progression-free survival was 10.2 months (95 % CI 5.7–12.6) for the letrozole group and 20.2 months (13.8–27.5) for the palbociclib plus letrozole group (HR 0.488, 95 % CI 0.319–0.748; one-sided *p* = 0.0004), which correlates to a doubled PFS in favor of the combination group. The overall survival is unknown and the follow-up is ongoing.

In addition to the benefit of palbociclib as initial endocrine-based therapy for metastatic hormone-receptor-positive breast cancer, PALOMA3 trial evaluated 521 patients with advanced hormone-receptor-positive, HER2-negative advanced breast cancer that had relapsed or progressed during prior endocrine therapy. Patients were randomly assigned in a 2:1 ratio to receive palbociclib and fulvestrant or placebo and fulvestrant [9]. PALOMA3 study concluded that palbociclib with fulvestrant resulted in longer progression-free survival and a relatively higher quality of life than fulvestrant alone in patients with advanced hormone-receptor-positive breast cancer that had progressed during prior endocrine therapy. The median progression-free survival was 9.2 months with palbociclib-fulvestrant and 3.8 months with placebo-fulvestrant (*p* < 0.0001, 95 % CI).

Consistent benefit from palbociclib was seen in all subgroups analyzed, with similar benefit in progression-free survival adding palbociclib in both premenopausal and postmenopausal women. Translational research for markers that might predict which group of patients benefits most is ongoing.

In addition to the favorable outcome from the clinical trials using palbociclib, other small molecule inhibitors of CDK4/6 are being studied in hope for developing more potent agents. In preclinical models, LEE011 (ribociclib) has demonstrated a dose-dependent antitumor activity that tracks well with CDK4/6 inhibition [10, 11]. Ribociclib is currently being evaluated in HR-positive breast cancer with letrozole and PI3K inhibitor BYL719 [12, 13]. A phase III study evaluating the combination of ribociclib with letrozole in HR-positive, HER2-negative breast cancer is ongoing (MONALESSA-2, NCT01958021). Other phase III studies investigating ribociclib in combination regimens for the treatment of women with HR-positive, HER2-negative advanced breast cancer are ongoing (MONALEESA-3, NCT02422615; [14]).

Abemaciclib (LY2835219) is another selective oral CDK4/6 inhibitor that is being developed in preclinical settings and clinical trials [15–17]. The combination of abemaciclib plus fulvestrant was evaluated in a small study (*n* = 13), which showed that combination therapy was well tolerated [18]. Several studies are planned which include a phase III randomized double-blind placebo-controlled trial of nonsteroidal aromatase inhibitor with or without abemaciclib in previously untreated advanced hormone-sensitive breast cancer (MONARCH 3, NCT02246621) [19]. A randomized double-blind placebo-controlled phase III study will compare the combination of abemaciclib with fulvestrant versus fulvestrant alone (MONARCH 2, NCT02107703) to investigate the benefit of abemaciclib in combination with endocrine therapy with a primary endpoint of PFS.

Future directions also include using the CDK4 and CDK6 inhibitors in the adjuvant and neoadjuvant therapy settings. A neoadjuvant trial investigating the combination of abemaciclib and aromatase inhibitor in locally advanced ER-positive, HER2-negative breast cancer (neoMONARCH, NCT02441946) is ongoing.

The results of the PALOMA 1/TRIO 18 study led to accelerated FDA approval of palbociclib in combination with letrozole in first line advanced ER+/HER2-breast cancer. The doubled PFS benefit observed in this randomized trial was impressive, but it still is awaiting confirmation from the phase III PALOMA-2 study. The PALOMA 3 study clearly showed that adding palbociclib to fulvestrant resulted in substantially longer progression-free survival than fulvestrant alone in patients with advanced HR-positive and HER2-negative breast cancer that had progressed during prior endocrine therapy, regardless of menopause status. In addition, the combination therapy is associated with relatively higher quality of life than with fulvestrant alone. Neutropenia was the most common adverse event in patients receiving palbociclib with low incidence of febrile neutropenia in both treatment groups. Although the combination of aromatase inhibitor with fulvestrant or everolimus remain excellent treatments for this group of patients [1, 20], PALOMA 3 offers an excellent alternative therapy for both premenopausal and postmenopausal patients who had progressed with prior endocrine therapy. Palbociclib and other CDK4/6 inhibitors being developed offer a new era of treatment for hormone-receptor-positive advanced breast cancer.
